# Low-dose lung CT: Optimizing diagnostic radiation dose – A phantom study

**DOI:** 10.1016/j.ejro.2024.100614

**Published:** 2024-11-24

**Authors:** Michael Zellner, Sebastian Tschauner, Mathias S. Weyland, Peter Eggenberger Hotz, Stephan Scheidegger, Christian J. Kellenberger

**Affiliations:** a University Children’s Hospital Zürich, Department of Diagnostic Imaging, Zurich, Switzerland; b University Children’s Hospital Zürich, Children’s Research Centre, Zurich, Switzerland; c Medical University of Graz, Department of Radiology, Division of Paediatric Radiology, Graz, Austria; d Cantonal Hospital Aarau AG, Applied Complex Systems Science, Aarau, Switzerland; e University of Applied Sciences Zurich, Zurich, Switzerland

**Keywords:** Tomography, X-Ray Computed, Low dose, Diagnostic imaging, Lung, Radiation

## Abstract

**Background/purpose:**

To investigate a quantitative method for assessing image quality of low dose lung computed tomography (CT) and find the lowest exposure dose providing diagnostic images.

**Methods:**

Axial volumetric lung CT acquisitions (256 slice scanner) were performed on three different sized anthropomorphic phantoms at different dose levels. The maximum steepness of sigmoid curves fitted to line density profiles was measured at lung-to-pleura interfaces. For each phantom, image sharpness was calculated as the median of 468 measurements from 39 different locations. Diagnostic image quality for the adult and paediatric phantom was rated by three radiologists using 4-point Likert scales. The image sharpness cut-off for obtaining adequate image quality was determined from qualitative ratings.

**Results:**

Adequate diagnostic image quality was reached at a median steepness of 713 HU/mm in the adult phantom with a corresponding CTDIvol of 0.14 mGy and an effective dose of 0.13 mSv at a dose level of 100 kVp and 10 mA. In the paediatric phantom diagnostic image quality was reached at a median steepness of 1139 HU/mm with a corresponding CTDIvol of 0.13 mGy and an effective dose of 0.08 mSv at a dose level of 100 kVp and 10 mA.

**Conclusions:**

Determination of image sharpness on line density profiles can be used as quantitative measure for image quality of lung CT. Sufficient-quality lung CT can be achieved at effective radiation doses of 0.13 mSv (adult phantom) and 0.08 mSv (paediatric phantom). These findings suggest that substantial dose reduction is feasible without compromising diagnostic accuracy.

## 1 Introduction

Computed tomography (CT) is the largest source for general and medical radiation exposure worldwide. The radiation dose delivered to patients in CT examinations is a public health concern [1], [2]. Radiation protection principles like “as low as reasonably achievable“ (ALARA) [3] and campaigns such as Image Gently [4] have led toward a better understanding of radiation exposure and have raised community awareness. Several studies have shown increased cancer risk in children who underwent CT and thus the importance of paediatric CT radiation protection [5], [6]. The technical dose reduction methods are X-ray spectral beam shaping (e.g. tin filtering), detector efficiency improvements, and new image reconstruction methods including iterative reconstruction algorithms and artificial intelligence methods [7], [8].

Modern multidetector row CT devices allow helical and axial scanning. In wide detectors of up to 16 cm, axial scanning is possible without table movement and is therefore beneficial in paediatric imaging [9]. In recent literature axial volumetric scan mode is described as the most dose efficient scan mode [10]. Photon counting detector CT is rapidly emerging as a crucial modality in lung imaging, offering enhanced spatial resolution and potentially improved dose efficiency compared to conventional CT systems [11], [12]. There is a wide variety of reported lung low-dose CT scan protocols in the current literature, with radiation doses ranging from minimal to significantly higher levels [11], [12], [13], [14]. This study emphasizes the importance of establishing a threshold for providing ultra-low-dose lung CT scans that still yield diagnostic-quality images. To account for the variability in anatomical sizes, three differently sized phantoms were used in this study, allowing for a more comprehensive analysis of dose optimization across a range of body types.

The aim of the study was to find the lowest exposure dose providing diagnostic lung CT images using a deep learning-based image reconstruction algorithm. In addition, we investigated and developed a quantitative method for assessing image quality of low dose lung CT and defined dose thresholds necessary for achieving fully diagnostic lung images.

## 2 Material and methods

### 2.1 CT acquisition and image reconstruction

This phantom study was conducted on a 256-row multidetector CT scanner (Revolution CT, GE Healthcare, Chicago, Illinois, USA). Three different-sized anthropomorphic phantoms were used in the study, including an adult whole-body phantom (PBU-60, Kyoto Kagaku, Kyoto, Japan), a paediatric chest phantom (PH-1C, Kyoto Kagaku, Kyoto, Japan) representing a 5-year-old, and a newborn whole-body phantom (PBU-80, Kyoto Kagaku, Kyoto, Japan).

First, the most dose efficient and fastest scan mode was sought by comparing different scan modes and collimations at a set CTDI_vol_ of 0.1 mGy.

Second, the phantoms were scanned with the axial wide detector mode at 13 different dose levels ranging from 70 kVp and 10 mA (70/10) to 140 kVp and 50 mA (140/50). The field of view (FOV) and scan range were adjusted to the phantom size, slice thickness was 0.625 mm and the rotation time 0.28 s (Table 1). The scan range included the entire lung/thorax, from apex to base. The scanning protocol involved dividing the adult phantom into three sequential 12 cm blocks (scan range 36 cm), whereas the paediatric and newborn phantoms were each scanned using a single axial block, with respective scan ranges of 16 cm and 10 cm. The images were reconstructed using deep learning image reconstruction (DLIR, TrueFidelity, GE Healthcare, Chicago, Illinois, USA) using medium strength denoising. Deep Learning Image Reconstruction (DLIR) offers significant advantages over traditional iterative methods, including enhanced image quality through improved noise reduction and better contrast resolution, particularly at low radiation doses [15].

**Table 1:** Scanner settings.

**GE Revolution CT Scanner**	
Detector rows, *N*	256
Slice collimation, *mm*	0.625
Detector width, *mm*	160
Used scan mode	axial
Rotation / exposure time, *sec*	0.280
Kernel	standard lung filter
Iterative reconstruction	DLIR medium
Pixel matrix	512×512
Reconstructed slice thickness, *mm*	0.625 / 1.0 (MPR average)

### 2.2 Subjective image quality evaluation

The thin (0.625 mm) axial lung images (window width 1500, centre −500) of the adult and paediatric phantom were viewed on the institutional picture archiving and communication system (PACS; IDS7; Sectra Medical Systems, Linköping, Sweden). Three readers (C.J.K., S.T., and M.Z., with 31, 12, and 9 years of experience in paediatric lung CT), who were blinded to the scan settings and radiation dose, assessed overall image quality and five subjective image quality items on 4-point Likert scales.

Overall image quality was rated as 1- unacceptable quality (images do not allow diagnostic interpretation), 2- limited quality (images are adequate only for limited clinical interpretation due to high noise), 3- adequate quality (images are just adequate for diagnostic interpretation), 4- Higher than needed quality (images are much better than needed for interpretation: images with little or no noise).

The presence of streak artefacts was rated as 1- severe, 2- moderate, 3- mild, 4- none. The perceptible image noise/ reticular pattern was rated as 1- severe, 2- moderate, 3- mild, 4- none. The sharpness of the pleura (peripheral lung to soft tissue interface) was rated as 1- unacceptable, 2- significantly reduced with blurring of adjacent structures, 3- mildly reduced, 4- excellent sharpness. The sharpness of the lung structures was rated as 1- unacceptable, 2- significantly reduced with blurring of adjacent structures, 3- mildly reduced, 4- excellent sharpness. The visibility / detection of small lung structures (defined beforehand) 1- unacceptable, 2- significantly reduced, 3- mildly reduced, 4- excellent.

Furthermore, we analysed the average sum of streak artefacts, image noise, sharpness and visibility of structures for each adult and paediatric phantom scan.

No subjective image quality assessment was performed in the newborn whole body phantom because it did not include an insert showing the lung structures. Quantitative image quality evaluation was performed in the same way as in the adult and paediatric phantom.

### 2.3 Image sharpness

Image sharpness was quantified as a measure of quality, expressed as the steepness (HU/mm) of sigmoid curves from line density profiles at lung-to-pleura interfaces.

**Fig. 1:**
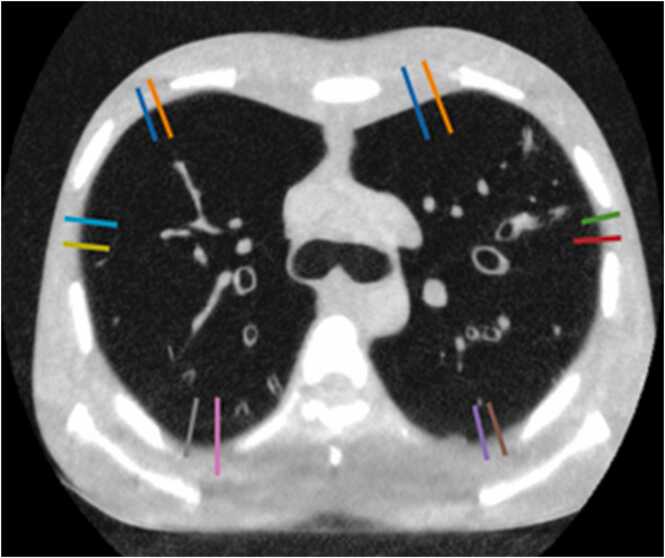
Example of locations at the lung to pleura interface mid chest in paediatric phantom used for measuring steepness on line density profiles.

To evaluate the sharpness of the lung-to-pleura transition, lines were drawn into the CT slice (image) I(x,y) (Fig. 1). Each line is characterized by its start and end coordinates, ($x_{\mathrm{begin}},y_{\mathrm{begin}}$) and ($x_{\mathrm{end}},y_{\mathrm{end}}$), respectively. For measuring the steepness, we used a logistic function

$$\hat{h}\left(l_{n};\theta_{0},\theta_{1},\theta_{\mathrm{begin}},\theta_{\mathrm{end}},\right)=\theta_{\mathrm{begin}}\;+\;\frac{\theta_{\mathrm{end}}-{\;\theta }_{\mathrm{begin}}}{1+\exp\left(-\theta_{1}\left(l_{n}-\theta_{0}\right)\right)}$$

to model the transition along a given line. The length $l_{n}$ is the distance of pixel $I_{n}$ from the start of the line. The function $\hat{h}$ has four parameters; $\theta_{0}$ is a location parameter that shifts $\hat{h}$ along the l-axis. $\theta_{1}$ affects the slope of $\hat{h}$. $\theta_{\mathrm{begin}}\mathrm{and}\theta_{\mathrm{end}}$ relate the asymptotic properties of the modelled transition: The line density profile starts at a given HU value (in the region before the line intersects with the material interface) and ends at another value. $\theta_{\mathrm{begin}}\mathrm{and}\theta_{\mathrm{end}}$ determine these two values. The steepness of the transition is found by evaluating $\left|\frac{\partial \hat{h}\left(l_{n};\theta_{0},\theta_{1},\theta_{\mathrm{begin}},\theta_{\mathrm{end}}\right)}{\partial l}\right|$ at the inflection point of the curve, which yields $\left|\frac{\theta_{1}\cdot \left(\theta_{\mathrm{end}}-{\;\theta }_{\mathrm{begin}}\right)}{4}\right|$. Absolute values were used since $\theta_{1}$ is negative in case of a falling density profile.

Parameter estimation was performed as follows: First, we used Bresenham's line algorithm [16] to find all pixels I(x,y) along a given line. For these pixels, we computed the distance $l_{n}$ from the start of the line. This yields pairs $\left(l_{n};h_{n}\right)$. ($\left(\tau -\tau_{0},L(\tau -\tau_{0})\right)$,) Where $h_{n}$ is the HU value corresponding to pixel I(x,y).Lastly, the four parameters $\hat{\theta }$ were estimated using the curve_fit function bundled with the python package scipy (version 1.3.3 running on python 3.8.5) and steepness of the profile was found by evaluating the steepness expression $\left|\frac{\theta_{1}\cdot \left(\theta_{\mathrm{end}}-{\;\theta }_{\mathrm{begin}}\right)}{4}\right|$.

A total of 39 different lung-to-pleura interfaces were measured at 3 levels (apical, mid-chest, basal) and 6 locations (anterior, lateral, and posterior) per side, yielding 468 measurements for each phantom and scan.

### 2.4 Signal-to-noise ratio and contrast-to-noise ratio

Besides image sharpness, signal-to-noise ratio (SNR) and contrast-to-noise ratio (CNR) were measured. One reader (MZ) manually placed circular regions of interest (ROI) in defined thin axial lung window slices (0.625 mm) for each phantom scan, ensuring consistent placement on the same slice to retrieve the mean Hounsfield Unit (HU) and standard deviation, with the latter representing image noise. The minimum allowed ROI area was 5000 pixels. The average of three repeated measurements in the trachea, the liver, and the air space anteriorly to the mid chest acted as basic objective image quality parameters. Based on these parameters, we calculated signal-to noise $\langle \;=\mathrm{SNR}=\;\frac{\mathrm{MEAN tissue}}{\mathrm{SD air}}\;\rangle$and contrast-to-noise $\langle \;=\mathrm{CNR}=\;\frac{\mathrm{MEAN}\left[\mathrm{MEAN}\left(\mathrm{liver},\mathrm{trachea}\right)\right]-\mathrm{MEAN air}}{\mathrm{SD air}}\;\rangle$ ratios.

### 2.5 Dose estimates

We collected the computed tomography dose indices (=CTDI_vol_, calibrated using a 32 cm phantom, mGy) and the dose length products $\langle \;=\mathrm{DLP}=\mathrm{CTDIvol}*\mathrm{scan lenth in cm}(\mathrm{mGy}*\mathrm{cm})\rangle$from the DICOM dose reports. Effective chest diameters were calculated as $\langle \;\sqrt[{2}]{\left[\mathrm{anteroposterior}\left(\mathrm{AP}\right)\;*\mathrm{mediolateral}\left(\mathrm{ML}\right)\mathrm{chest diameter}\right]}\;\rangle$, rounded to full centimeters. The effective diameters together with the size-dependent correction factors, derived from the publications by the American Association of Physicists in Medicine [17] and by Romanyukha et al. [18], served as calculation basis for size specific dose estimates (=SSDE, *mGy*) and effective doses (=ED, *mSv*), the latter calculated as DLP multiplied with the diameter-specific conversion factor [18].

### 2.6 Dose level thresholds for diagnostic image quality

In our study, we conducted a subjective assessment that included evaluations of overall image quality alongside the summation of five specific image quality items. Diagnostic image quality was defined as the cumulative score of these five assessments, requiring a total score of more than 14 and an overall quality score of at least 3 for both paediatric and adult phantoms. Subsequently, we computed a quality score and investigated its correlation with the dose levels. Furthermore, we established specific diagnostic doses for each phantom using the median steepness of line density profiles.

### 2.7 Statistical analysis

We analysed the collected data in SPSS Statistics Version 26 (IBM Corp., Armonk, NY, USA) by computing descriptive statistics. Intraclass correlation coefficients (ICC) served as measures of inter-observer reliability in terms of ICC(3,k) two-way mixed, average measures. ICC values below 0.5 refer to poor, 0.5–0.75 moderate, 0.75–0.9 good, and higher than 0.9 to an excellent reliability [19].

## 3 Results

### 3.1 Subjective image quality

All raters consistently rated the overall image quality of the adult phantom as at least adequate when using scan settings of 100 kV and 10 mA, resulting in a CTDIvol of 0.14 mGy, a SSDE of 0.20 mGy, and an effective dose of 0.13 mSv. Similarly, all readers found the image quality of the paediatric phantom to be at least adequate using scan settings of 100 kV and 10 mA, which yielded a CTDIvol of 0.13 mGy, a SSDE of 0.19 mSv, and an effective dose of 0.08 mSv. Similar overall image quality results with a higher effective dose were observed using scan settings of 80 kV and 20 mA in both phantoms.

In both the adult and paediatric phantom, the average sum of the five subjective image quality criteria, consistently indicated at least adequate overall image quality (score more than 14) with correlating scan settings starting at 100 kV and 10 mA.

**Fig. 2:**
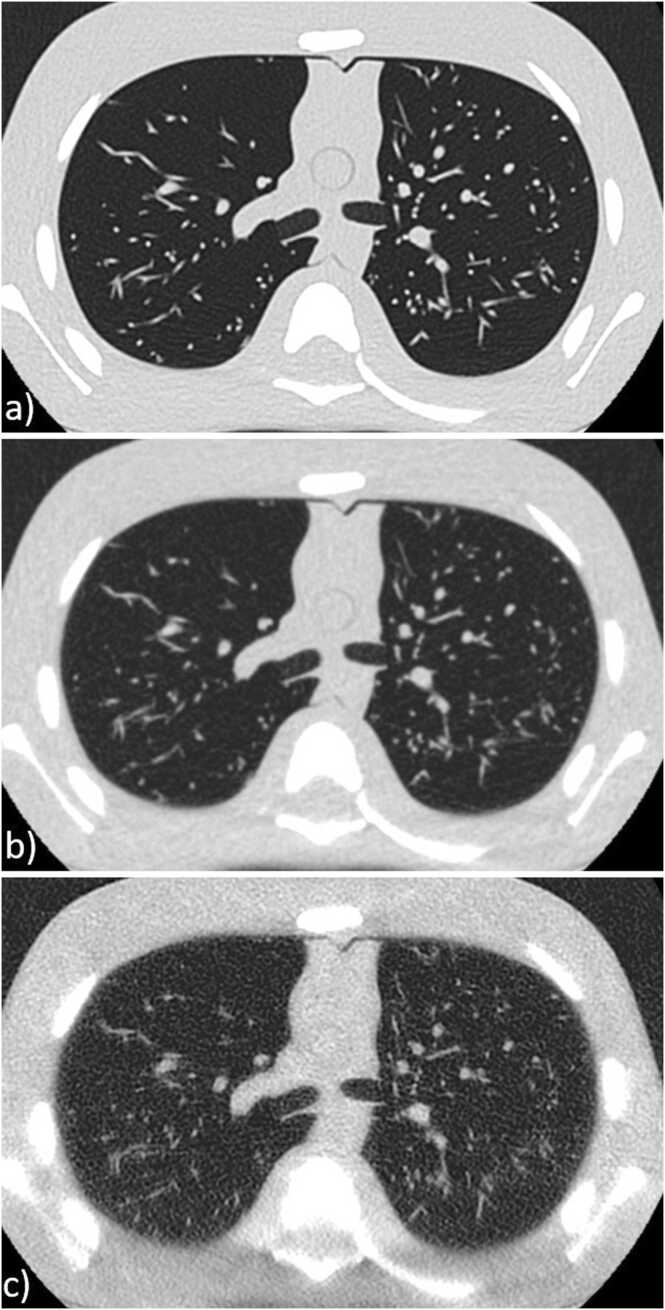
Paediatric phantom scan: a) scanned with 140 kVp and 50 mAs (higher than needed quality), b) scanned with 100 kVp and 10 mAs (diagnostic quality) and c) scanned with 70 kVp and 10 mAs (not diagnostic quality).

Fig. 2(a-c) represents a visual example of a scan of the paediatric phantom, illustrating the distinctions between diagnostic, non-diagnostic, and adequate diagnostic quality.

For subjective image quality rating per reader for both phantom scans see Table 2.

**Table 2:** Subjective quality assessment of lung images from the adult and paediatric phantom by 3 readers (CK, MZ, ST).

kVp kilovoltage (peak), mAs tube current time product (milliampere x seconds), CTDIvol computed tomography dose index volume in mGy, ssde size specific dose estimate in mGy, ed effective dose in mSv. Image quality is coded from yellow (not diagnostic) to dark green (higher than needed quality).

ICC was 0.991 (95 % confidence interval from 0.982 to 0.997) for the adult phantom and 0.992 (95 % confidence interval from 0.984 to 0.997) for the paediatric phantom meaning excellent agreement on quality rating of adult and paediatric phantoms [19].

The lowest exposure setting (70 kV and 10 mA) in adult and paediatric phantom was rated by 2 readers (ST and MZ) as unacceptable quality.

### 3.2 Objective image quality

A CNR of 12.29 and a SNR of 11.43 in the adult phantom (scan setting 100 kV and 10 mA) and a CNR of 15.16, SNR of 15.45 in the paediatric phantom (scan setting 100 kV and 10 mA) showed adequate image quality. The median steepness at these settings were 713 HU/mm in the adult and 1139 HU/mm in the paediatric phantom (Table 3).

**Table 3:** Scan settings and objective image quality from all phantom scans.

**ID**	**Phantomtype**	**kVp**	**mA**	**mAs**	**CTDIvol (mGy)**	**CNR**	**SNR**	**Median steepness (HU/mm)**
1	Adult phantom	140	50	14.00	1.59	18.64	17.27	5324.49
2	Adult phantom	140	40	11.20	1.20	17.25	16.06	3995.02
3	Adult phantom	140	30	8.40	0.95	16.35	15.22	2673.91
4	Adult phantom	140	20	5.60	0.63	15.58	14.46	2195.73
5	Adult phantom	140	10	2.80	0.32	13.19	12.19	1297.25
6	Adult phantom	120	20	5.60	0.44	14.15	13.14	1590.85
7	Adult phantom	120	10	2.80	0.22	13.03	11.99	885.54
8	Adult phantom	100	20	5.60	0.27	13.40	12.33	937.71
9	Adult phantom	100	10	2.80	0.14	12.30	11.43	713.28
10	Adult phantom	80	20	5.60	0.14	11.24	10.47	572.30
11	Adult phantom	80	10	2.80	0.07	9.78	9.85	474.95
12	Adult phantom	70	20	5.60	0.09	10.51	10.68	397.29
13	Adult phantom	70	10	2.80	0.04	7.17	8.39	257.97
14	Paediatric phantom	140	50	14.00	1.43	24.30	24.36	3451.98
15	Paediatric phantom	140	40	11.20	1.14	24.91	25.01	3271.14
16	Paediatric phantom	140	30	8.40	0.86	22.08	22.16	3070.61
17	Paediatric phantom	140	20	5.60	0.57	17.96	17.99	2681.34
18	Paediatric phantom	140	10	2.80	0.29	16.12	16.08	1962.56
19	Paediatric phantom	120	20	5.60	0.40	15.06	15.25	2318.57
20	Paediatric phantom	120	10	2.80	0.20	14.96	15.15	1505.04
21	Paediatric phantom	100	20	5.60	0.25	14.88	15.24	1775.20
22	Paediatric phantom	100	10	2.80	0.13	15.16	15.46	1139.83
23	Paediatric phantom	80	20	5.60	0.13	12.89	13.46	1195.68
24	Paediatric phantom	80	10	2.80	0.07	11.84	12.26	685.00
25	Paediatric phantom	70	20	5.60	0.09	11.64	12.27	743.76
26	Paediatric phantom	70	10	2.80	0.04	9.63	10.33	651.23
27	Neonate phantom	140	50	14.00	1.19	75.46	74.09	2478.05
28	Neonate phantom	140	40	11.20	0.95	66.58	65.45	2477.14
29	Neonate phantom	140	30	8.40	0.71	55.17	54.02	2455.41
30	Neonate phantom	140	20	5.60	0.48	45.74	45.03	2601.04
31	Neonate phantom	140	10	2.80	0.24	36.22	35.39	2399.42
32	Neonate phantom	120	20	5.60	0.33	42.33	41.80	2386.29
33	Neonate phantom	120	10	2.80	0.17	30.80	30.34	2084.18
34	Neonate phantom	100	20	5.60	0.21	34.33	33.90	2375.71
35	Neonate phantom	100	10	2.80	0.10	27.07	26.99	2089.35
36	Neonate phantom	80	20	5.60	0.11	27.52	27.54	2082.85
37	Neonate phantom	80	10	2.80	0.06	21.06	21.04	1690.77
38	Neonate phantom	70	20	5.60	0.07	24.80	25.06	1824.99
39	Neonate phantom	70	10	2.80	0.04	18.91	19.19	1101.76

kVp kilovoltage (peak), mAs tube current time product (milliampere x seconds), CTDIvol computed tomography dose index volume in mGy, CNR contrast to noise ratio, SNR signal to noise ratio.

### 3.3 Quantitative image quality to dose relation

**Fig. 3:**
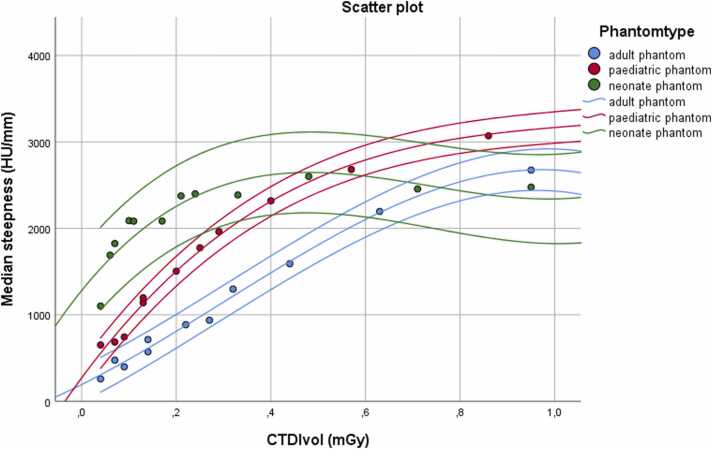
Scatter plot of the median steepness over CTDIvol with a fitted cubic regression line and a 95 % confidence interval showing the increase of sharpness with dose.

**Fig. 4:**
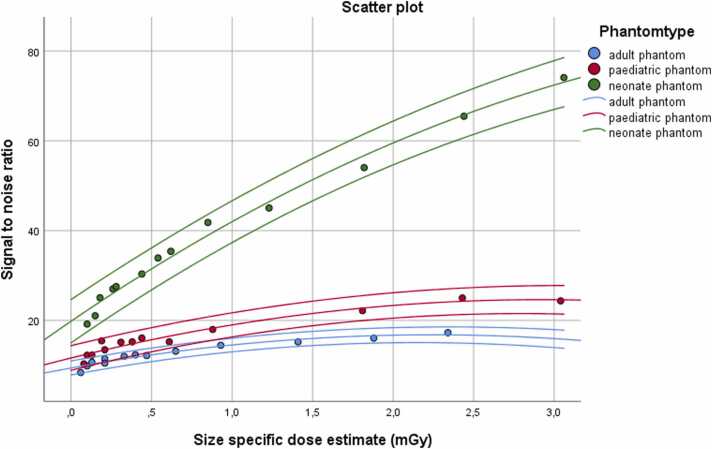
Scatter plot of signal to noise ratio over size specific dose estimate in mGy with a fitted quadratic regression line and a 95 % confidence interval showing a plateau of SNR at higher dose levels in the paediatric phantom.

Image sharpness/ steepness of line density profiles increased with higher CTDIvol in all phantoms with a steep increase at low dose and less increase at higher dose (Fig. 3). The dose to median steepness curves started to flatten above 0.2 mGy in the neonate phantom, above 0.4 mGy in the paediatric and above to 0.8 mGy in the adult phantom. Both SNR and CNR increased with higher dose in the adult and neonate phantom, with an initial steep increase and flattening of the SNR/CNR to dose curves above an SSDE of approximately 0.4 mGy (Fig. 4).

In summary, sufficient-quality lung CT images can be achieved at effective radiation doses of 0.13 mSv for the adult phantom (100 kV and 10 mA) and 0.08 mSv for the paediatric phantom (100 kV and 10 mA).

## 4 Discussion

In this study, we assessed image quality at different dose levels in three phantoms. We defined dose thresholds for diagnostic image quality based on qualitative and quantitative image quality measurements.

Especially in paediatric radiology, lowering the radiation dose while still achieving diagnostic-quality images is crucial. Children are more sensitive to radiation exposure due to their developing tissues and longer life expectancy, making it essential to minimize doses without compromising diagnostic accuracy. Implementing techniques that optimize image quality at reduced doses not only enhances patient safety but also aligns with the ALARA principle in medical imaging [20].

Subjective image quality interrater agreement was good to excellent. The most experienced paediatric radiologists rated fewer phantom scans as unacceptable quality. This underscores the challenge of accurately determining the appropriate radiation dose for paediatric lung imaging. The selection of optimal exposure and reconstruction parameters is expected to remain a topic of discussion and influenced by individual preferences. The development of a quantitative approach for defining image quality appears to hold significant importance [21]. The penetration capability of the beam is influenced by the kVp, which also plays a crucial role in determining beam quality. Increased kVp results in greater penetration [22]. Our results suggest, that 100 kVp is necessary to penetrate the chest wall. Therefore, lowering the mA to the lowest possible setting (e.g. 10 mA on our scanner), seems to be preferable to decreasing kVp for achieving low effective doses in high contrast lung imaging.

We achieved diagnostic imaging quality using a CTDIvol of 0.14 mGy, a SSDE of 0.20 mGy, an effective dose of 0.13 mSv in the adult phantom and a CTDIvol of 0.13 mGy, a SSDE of 0.19 mSv, and an effective dose of 0.08 mSv for the paediatric phantom. The U.S. diagnostic reference values given for chest CT are 15 times higher, with an SSDE of 3.0 mGy for a thoracic CT without contrast [23]. In the most recent literature using novel technology such as photon counting lung CT a SSDE of 0.45 +- 0.14 mGy in paediatric patients was achieved [24]. Our study indicates that similar doses can be achieved with conventional CT and that there may be a potential for achieving even lower doses with photon counting. A larger study by Demb et al. analyzed the radiation doses of 12,529 patients undergoing lung cancer screening across various institutions in the USA, reporting a mean CTDIvol of 2.4 ± 2 mGy [14]. Similarly, a study by Woeltjen involving 29 adult patients achieved an average CTDIvol of 2.5 ± 1.1 mGy [12]. In contrast, a study by Tschauner et al. found a CTDIvol of 0.26 ± 0.14 mGy in 23 paediatric patients [13]. These studies illustrate the significant variance in radiation dose ranges internationally, highlighting the relevance and importance of our phantom study in the context of dose optimization and the need for standardized practices.

In addition, our study confirms that increasing the exposure dose above a certain threshold does not lead to a proportional increase in image quality. Maximum subjective overall image quality ratings were reached at 0.3 mGy CTDIvol. Quantitative image quality measures (SNR, CNR and image sharpness/steepness of line-density profiles) increased steeply at low dose levels but tended to plateau above 0.6 mGy CTDIvol. These findings indicate that raising the dose to very high levels results in diminishing returns in terms of image quality improvement.

Deep learning-based image reconstruction techniques often introduce complex transformations to the original data, aiming to enhance image quality and features. These transformations can lead to variations in noise distribution and contrast, making the straightforward application of SNR and CNR less appropriate [8], [13]. This shows the importance of having additional quantitative parameters such as steepness of line density profiles for evaluating image quality.

While our study provides valuable insights into dose optimization for lung CT imaging, it is important to acknowledge that it is a phantom study utilizing Kyoto phantoms. One of the primary limitations of this approach is that the findings may not fully translate to clinical practice, as phantoms do not completely replicate the complex anatomy and physiological variations of actual patients. Additionally, although the lung inserts in the Kyoto phantoms are designed to have Hounsfield unit values similar to those of human lung tissue, they may not accurately reflect the variations in tissue density and composition found in real patients. To address this limitation, we measured the lung-to-pleura surface in our assessments, which helps to better reflect the relationship between the lung tissue and surrounding structures. However, it is important to recognize that despite these efforts, the inherent differences between phantoms and actual patient anatomy may still influence imaging characteristics and outcomes.

Therefore, while our results offer a foundation for understanding the relationship between radiation dose and image quality, further studies involving actual patients will be necessary to validate our findings and confirm their clinical applicability. The inability to examine the neonate phantom's lung structures due to their absence poses another limitation to our paper.

## 5 Conclusions

Sufficient-quality lung CT can be achieved on contemporary scanners equipped with deep learning-based image reconstruction at effective radiation doses of 0.1 mSv and SSDE of 0.2 mGy in adult and paediatric phantoms.

Line density profiles of lung to pleura interfaces can provide a quantitative method for evaluating the sharpness and image quality of lung CTs.

## Ethics approval

Ethical approval was deemed unnecessary for this phantom study, as it did not involve any participation from actual patients

## Funding

The authors declare that no funds, grants, or other support were received during the preparation of this manuscript.

## CRediT authorship contribution statement

**Michael Zellner:** Writing – review & editing, Writing – original draft, Visualization, Validation, Software, Resources, Methodology, Investigation, Formal analysis, Data curation, Conceptualization. **Christian J Kellenberger:** Writing – review & editing, Supervision, Project administration, Investigation, Formal analysis, Data curation. **Sebastian Tschauner:** Validation, Methodology, Formal analysis, Conceptualization. **Mathias S Weyland:** Software, Methodology, Investigation, Formal analysis, Data curation. **Peter Eggenberger Hotz:** Software, Methodology, Data curation. **Stephan Scheidegger:** Software, Methodology, Formal analysis, Data curation.

## Declaration of Competing Interest

The authors have no relevant financial or non-financial interests to disclose.

## Data Availability

A complete dataset of all measurements is available as a supplement (Dataset 1) to this manuscript. Image data is stored at the author’s institution.
